# Chemical Changes Under Heat Stress and Identification of Dendrillolactone, a New Diterpene Derivative with a Rare Rearranged Spongiane Skeleton from the Antarctic Marine Sponge *Dendrilla antarctica*

**DOI:** 10.3390/md23010010

**Published:** 2024-12-28

**Authors:** Andrea Prófumo, Conxita Avila, Adele Cutignano

**Affiliations:** 1 Departament de Biologia Evolutiva, Ecologia i Ciències Ambientals (BEECA), Facultat de Biologia, Universitat de Barcelona (UB), Av. Diagonal 643, 08028 Barcelona, Catalonia, Spain; conxita.avila@ub.edu; 2 Institut de Recerca de la Biodiversitat (IRBio), Universitat de Barcelona (UB), 08028 Barcelona, Catalonia, Spain; 3 Consiglio Nazionale delle Ricerche (CNR), Istituto di Chimica Biomolecolare (ICB), Via Campi Flegrei 34, 80078 Pozzuoli, Napoli, Italy; acutignano@icb.cnr.it

**Keywords:** chemical ecology, global change, Porifera, terpenoids, marine natural products, metabolomics

## Abstract

The waters around the western Antarctic Peninsula are experiencing fast warming due to global change, being among the most affected regions on the planet. This polar area is home to a large and rich community of benthic marine invertebrates, such as sponges, tunicates, corals, and many other animals. Among the sponges, the bright yellow *Dendrilla antarctica* is commonly known for using secondary diterpenoids as a defensive mechanism against local potential predators. From the dichloromethane extract of sponge samples from Deception Island collected in January 2023, we isolated a novel derivative with an unusual β-lactone diterpene skeleton here named dendrillolactone (**1**), along with seven previously described diterpenes, including deceptionin (**2**), a gracilane norditerpene (**3**), cadlinolide C (**4**), a glaciolane norditerpene (**5**), membranolide (**6**), aplysulphurin (**7**), and tetrahydroaplysulphurine-1 (**8**). Here, we also report our studies on the changes in the chemical arsenal of this sponge by slow temperature increase in aquaria experiments. Despite being a species capable of inhabiting volcanically active areas, with frequent water temperature fluctuations due to the existing fumaroles, the results show that diterpenes such as deceptionin, cadlinolide C, membranolide, and tetrahydroaplysulphurin-1 seem to be susceptible to the temperature increase, resulting in a trend to higher concentrations. However, temperatures above 4 °C severely affected sponge metabolism, causing its death much earlier than expected. Further research on the roles of these natural products in *D. antarctica* and their relationship to the sponge’s resilience to environmental changes should help to better understand the defensive mechanisms of Antarctic marine benthos in the context of global change.

## 1. Introduction

Polar regions are suffering accelerated warming, and their ecosystems are becoming more vulnerable to global change over time [1,2,3,4]. In the Southern Ocean, despite the Antarctic Circumpolar Current (ACC) playing a crucial role in maintaining Antarctica’s thermal isolation, the western Antarctic Peninsula (WAP) is experiencing faster warming than other parts of Antarctica [4,5,6,7]. The fragile marine ecosystems inhabiting these areas, home to unique biodiversity, are currently facing warmer seawater temperatures along with the melting of extensive ice sheets, ice shelf collapse, and the continuous reduction in annual sea ice duration [3,6,8,9]. All these factors may reduce the resilience of these Antarctic marine organisms and the ecosystems they live in [10].

Marine sponges are a key animal group in the Antarctic benthos, representing one of the largest groups in percentages of both abundance and biomass within the dominant benthic communities [11,12,13,14,15]. Sponges form complex three-dimensional communities that provide habitat and refuge and facilitate the recruitment of many invertebrates and fish [15,16]. Moreover, sponges are crucial to marine ecosystems as they contribute to the accumulation of particulate material from the water column through filtration [17,18]. Sponges also host photosynthetic symbionts as well as heterotrophic bacteria, which facilitate the recycling of remineralised nutrients back into the marine environment [18,19,20]. It is estimated that more than 400 species of sponges have been identified in Antarctica, 44% of which are endemic, and over 70% belong to the class Demospongiae [16,21]. Demospongiae exhibit a wide bathymetric range, from shallow waters to abyssal zones, and live in all areas of the planet [13,22,23]. Many demosponges possess chemical defences against potential predators, including terpenoids, alkaloids, peptides, and macrolides [24,25,26,27,28,29,30,31].

The demosponge *Dendrilla antarctica* Topsent, 1905 (previously *Dendrilla membranosa*), is one of the most studied marine organisms in Antarctica, exhibiting a broad depth range of approximately 5–550 m [32,33]. In fact, *D. antarctica* is commonly found inhabiting many shallow coastal zones of Antarctica, notably along the WAP [34], although it has recently been reported also in South America [30]. Recent genetic studies have revealed a subtle population structure and signals of local adaptation, suggesting that *D. antarctica* is undergoing divergent selection despite gene flow between populations, especially in response to environmental conditions across different regions of the WAP [35]. *D. antarctica* is renowned for its chemical diversity, particularly due to a wide array of natural products, including numerous oxidised diterpenes. These compounds have been studied extensively over several decades, leading to important findings that highlight their broad ecological roles and potential pharmacological applications [30,36,37,38,39,40,41,42,43,44,45,46,47,48,49,50,51,52,53,54,55,56,57].

*D. antarctica* can display different chemical profiles, even within the same population, and its chemistry may change with subtle high-temperature increments [50]. Additionally, further variability in the metabolic profile of this sponge has been related to predation pressure by the amphipod communities inhabiting nearby areas [49]. Given the crucial role of chemical ecology in these communities [46,48,49,50,58,59,60], the defensive function of *D. antarctica* metabolites provides an ecological advantage that can be threatened by global change [50]. More specifically, the increasing sea water temperatures in Antarctic regions, such as the WAP, could affect the production of chemical compounds, thus decreasing the sponge’s defence against potential predators, competitors, pathogens, and others, or even making their survival harsh. Therefore, to further understand the changes in the chemical profile of *D. antarctica* with increasing sea water temperature, we aimed to evaluate the impact of slow heat stress on the terpene profile of the sponge *D. antarctica* and to further analyse the secondary metabolite profile of a population from the volcanic Deception Island (South Shetland Islands, Antarctica). In the frame of our metabolomic analysis, we identified and structurally characterised a novel terpene derivative exhibiting an unusual rearranged spongiane skeleton, which we named dendrillolactone (**1**), isolated from the specimens there collected. This research provides a deeper understanding of the potential impacts of global change on these Antarctic sponges. An understanding of global change impacts on marine benthic invertebrates is important not only for the management and conservation of Antarctic marine ecosystems but also for exploring the potential biotechnological applications of the sponges’ natural products. Overall, the results of this study should provide valuable insights into how environmental changes threaten both marine biodiversity and chemodiversity.

## 2. Results

### 2.1. Identification of D. antarctica Natural Products

**Figure 1:**
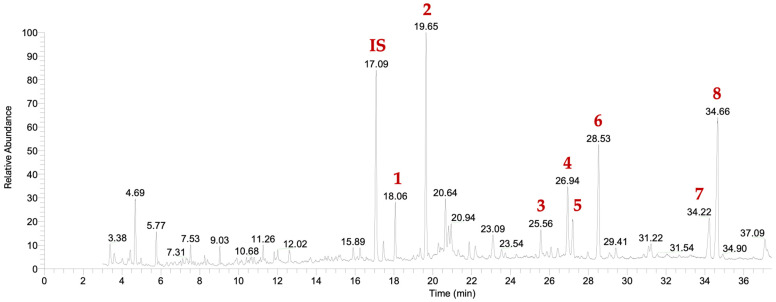
Representative GC-MS chemical profile of *D. antarctica* extracts: internal standard (IS). DDL—dendrillolactone (**1**); DCP—deceptionin (**2**); GRN—the gracilane norditerpene **3**; CLC—cadlinolide C (**4**); GLN—the glaciolane norditerpene **5**; MBN—membranolide (**6**); APS—aplysulphurin (**7**); and TTS—tetrahydroaplysulphurin-1 (**8**).

**Figure 2:**
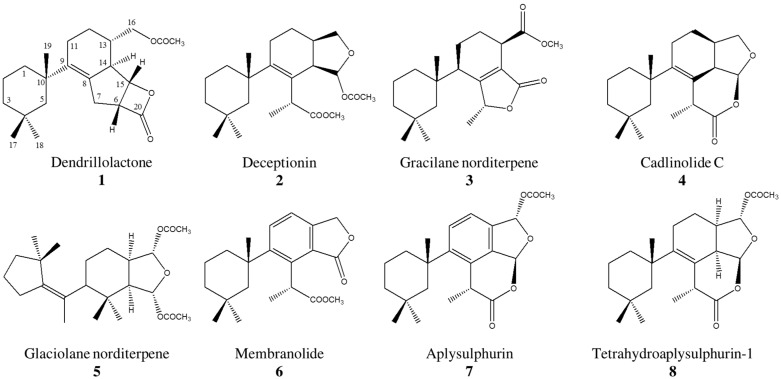
Diterpene metabolites **1**–**8** of *D. antarctica* from Deception Island (Antarctica).

We conducted a thorough analysis of the chemical composition of *D. antarctica* specimens (n = 15) collected at Deception Island, in the South Shetland Islands (Antarctica). The GC-MS data revealed eight main peaks in the extracts (Figure 1 and Figures S1–S8), which were tentatively attributed to terpenes. The identities of seven of these compounds were confirmed following their isolation from the crude extract and/or by comparison of the NMR and MS data with the literature. They were identified as the known deceptionin (DCP) [50] (**2**, RT = 19.65), the gracilane norditerpene **3** (GRN) [41] (RT = 25.56 min), cadlinolide C (CLC) [47] (**4**, RT = 26.94 min), the glaciolane norditerpene **5** (GLN) [52] (RT = 27.17 min), membranolide (MBN) [36] (**6**, RT = 28.53 min), aplysulphurin (APS) [53] (**7**, RT = 34.22 min), and tetrahydroaplysulphurin-1 (TTS) [61] (**8**, RT = 34.66 min) (Figure 1 and Figure 2). The spectroscopic and spectrometric data for the remaining compound (**1**) did not correspond to any known terpene and it was characterised as a novel diterpene derivative (Figures S9–S15). The new compound has been designated as dendrillolactone (**1**, DDL, RT = 18.06 min) (Figure 1, Figure 2 and Figures S1 and S9–S15) and it is an analogue of spongiolactone previously isolated from the sponge *Spongionella gracilis* [62].

### 2.2. Structural Characterisation of Dendrillolactone (**1**)

Compound **1** was isolated by HPLC and the molecular formula C_22_H_32_O_4_ was inferred by HRESI-MS of the M+Na^+^ adduct at *m*/*z* 383.2199 and by ^13^C-NMR data (Figures S9 and S11). The ^1^H NMR spectrum resembled those of other terpenes isolated from *D. antarctica* extracts (Figure S10). In particular, it showed four singlet signals of methyl groups: three of these at δ 0.80 (C-17, 26.2 ppm), δ 0.87 (C-18, 33.1 ppm), and δ 0.99 (C-19, 30.8 ppm) on the basis of the observed 2D-NMR correlations were located on a cyclohexane ring, whose complete assignment was accomplished by spectroscopic data analysis (Figure 2, Table 1, and Figures S12–S15); the last methyl singlet at δ 2.08 was attributed to an acetyl group located on C-16. A deep interpretation of homonuclear COSY and TOCSY data (Figures S12 and S13) together with HSQC and HMBC hetero-correlations (Figures S14 and S15) allowed for elucidating an extended spin system across a tricyclic system, including a cyclohexene with an acetoxymethyl branch, a five-membered carbocycle, and a β-lactone (Table 1). The connection between the two cyclic substructures was secured by the key HMBC correlations H_3_-19/C-9 and H_2_-5/C-9 (Table 1). An accurate survey of the literature revealed that the NMR spectroscopic data of the planar carbon skeleton of **1** were very similar to those reported for the spongiolactone from *S. gracilis* [62] (Figures S10–S15). The only difference was attributable to the ester derivative of the primary hydroxyl function at C-16, which in spongiolactone was an isovalerate in place of an acetate as in **1**. The NOE experiments suggested for **1** the same relative stereochemistry as reported for spongiolactone. Furthermore, the values of *J* couplings measured for the ring protons H-6, H_2_-7, H-14, and H-15 were perfectly in agreement with a synthetic derivative showing the same relative configuration of the tricyclic system [63]. The positive value of the optical rotation (α_D_ = +70.0) measured for the new derivative was close to that reported for spongiolactone (α_D_ = +67.6) [62] and the stereostructure is proposed as depicted in **1**.

### 2.3. Comparative Analysis of Control Groups: Natural vs. Aquarium Conditions

**Figure 3:**
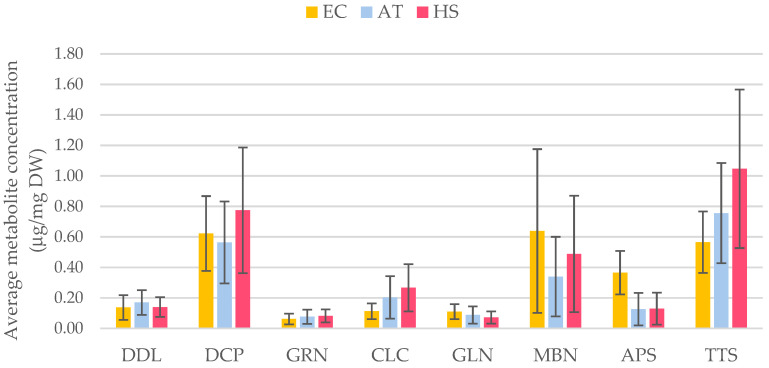
Quantification of main diterpenes in the control and treatment groups. The values of all the molecules are reported as the mean μg/mg of dry sponge weight (DW) ± SD (n = 5). EC—environmental control; AT—aquarium temperature control; and HS—heat stress treatment. DDL—dendrillolactone (**1**); DCP—deceptionin (**2**); GRN—the gracilane norditerpene **3**; CLC—cadlinolide C (**4**); GLN—the glaciolane norditerpene **5**; MBN—membranolide (**6**); APS—aplysulphurin (**7**); and TTS—tetrahydroaplysulphurin-1 (**8**).

The comparison of the aquarium control (AT) specimens to those analysed directly from their natural habitat (EC) indicates that there could be some effect of the aquarium environment, affecting the metabolic profiles of natural products (Figure 3). However, although this effect was observed in the variability of the concentration of all the molecules at different levels (Table S2 and Figure 3), these changes are not statistically significant in the Wilcoxon pairwise tests (*p* > 0.05). Thus, these results indicate that the chemical profile of AT is not significantly different from that of EC but shows a wide variation. More specifically, the CLC (**4**) (0.11 to 0.20 µg/mg DW) and TTS (**8**) (0.57 to 0.76 µg/mg DW) contents were higher in the AT group, while in the same group the concentration of DCP (**2**) (0.62 to 0.56), MBN (**6**) (0.64 to 0.34 µg/mg DW), and APS (**7**) (0.37 to 0.13 µg/mg DW), exhibited a decline. On the other hand, the concentrations of DDL (**1**) (0.14 to 0.17 µg/mg DW), GRN **3** (0.06 to 0.08 µg/mg DW), and GLN **5** (0.11 to 0.09 µg/mg DW) remained quite stable in the aquarium control specimens. The overall disparity in metabolite levels between the natural environment and the aquarium controls was quantified using the SIMPER test and indicated that change was driven primarily by variations in MBN (**6**), TTS (**8**), and APS (**7**) concentrations (av. dissimilarity = 10.91; Table 2).

### 2.4. Analysis of Terpene Metabolite Responses to Induced Heat Stress

The thermal stress seemed to indicate that there was an impact on the metabolic profiles, with differences in the compound concentrations between the AT and heat stress (HS) temperature. A comparative analysis of the chemical profiles revealed an average increase in the concentrations of secondary metabolites in the HS group (Figure 3 and Table S2). Specifically, the DCP (**2**) increased from 0.56 to 0.77 µg/mg DW, CLC (**4**) from 0.20 to 0.27 µg/mg DW, MBN (**6**) from 0.34 to 0.49 µg/mg DW, and TTS (**8**) from 0.76 to 1.05 µg/mg DW. In contrast, molecules like DDL (**1**) (0.17 to 0.14 µg/mg DW), GRN (**3**) (0.08 µg/mg DW), GLN **5** (0.09 to 0.07 µg/mg DW), and APS (**7**) (0.13 µg/mg DW) showed no notable differences in their concentrations despite thermal stress exposure.

When comparing the concentrations between EC and HS, different results were observed: the DCP (**2**) increased from 0.62 to 0.77 µg/mg DW, CLC (**4**) from 0.11 to 0.27 µg/mg DW, and TTS (**8**) from 0.57 to 1.05 µg/mg DW, whereas the MBN (**6**) decreased from 0.64 to 0.49 µg/mg DW and APS (**7**) from 0.37 to 0.13 µg/mg DW (Figure 3 and Table S2). Additionally, the DDL (**1**) remained stable at 0.14 µg/mg DW, while the GRN (**3**) ranged from 0.06 to 0.08 µg/mg DW and GLN (**5**) from 0.11 to 0.07 µg/mg DW. Despite all these variations, the Wilcoxon pairwise tests for AT vs. HS as well as for EC vs. HS were not statistically significant (*p* > 0.05). Moreover, the PERMANOVA analysis, which included all three groups (AT, EC, and HS), was not statistically significant either (*p* > 0.05).

The SIMPER test indicated an 88.77% average similarity within the HS group, with TTS (**8**), DCP (**2**), and MBN (**6**) being the primary contributors, accounting for a cumulative 47.34% (Table 3). Similarly, the AT group samples exhibited an 87.61% similarity, with the same molecules contributing 44.76% cumulatively. Despite the internal similarities within the groups (Table 3), a significant dissimilarity between the AT and HS was observed, driven primarily by molecules like TTS (**8**), MBN (**6**), and DCP (**2**) (Table 2). These molecules contributed the most to the dissimilarity, accounting for a cumulative 53.12% of the total differences between the groups. In particular, TTS (**8**) alone contributed 24.06% to the overall dissimilarity, highlighting its role as a key indicator between the two groups. Furthermore, the dissimilarity between the EC and HS was also driven by the contributions of MBN (**6**), TTS (**8**), and APS (**7**), with a cumulative contribution of 52.93%, resulting in an average dissimilarity of 11.27 for the comparisons. This pattern suggests that, while intra-group variability remains low, inter-group differences are largely defined by a few dominant molecules, such as TTS (**8**).

It is worth mentioning that the experiment finished at 4 °C after 10 days, instead of the planned 5 °C after two weeks, due to the low viability of the sponge samples in the HS treatment. The specimens showed signs of physiological stress and degeneration when the temperature reached 4 °C. The experiment was then considered finished, and all the sample treatments were frozen.

## 3. Discussion

### 3.1. Natural Products of D. antarctica from Deception Island and Identification of Dendrillolactone (**1**)

This study describes a new diterpene derivative within the chemical profile of *D. antarctica* from Deception Island, here named dendrillolactone (**1**). This compound was not or barely detected in previous collections of the sponge from the same area although not from the same specific sampling site. The new metabolite shows a rare rearranged spongiane skeleton with a fused β-lactone ring previously reported only in another compound from the sponge *S. gracilis*, namely, spongiolactone [62], and successively in its 3′-nor-derivative from *Spongionella sp*. [64]. Bioactivity studies on spongiolactones and synthetic congeners revealed selective cytotoxic properties against K562 human chronic myelogenous leukaemia cells [63,65]. However, the cellular targets of spongiolactones are poorly understood and it seems that the complex, rigid skeleton may be responsible for multiple enzymatic targets offering an intriguing poly-pharmacological profile [65]. This anticipates also for DDL (**1**) promising biological properties that are worth exploring.

Indeed, marine sponges (Porifera) are organisms particularly well known and valued worldwide due to their richness in biologically active chemical compounds that may serve as effective defence mechanisms, as mentioned above. Also, while many of these bioactive molecules are still in clinical trials, some others already have notable biomedical applications against cancer [54,66] or other diseases [67]. In Antarctica, research related to the isolation of compounds from marine sponges is gradually gaining momentum despite the difficulties involved (sampling periods, sample collection, experiments implementation, etc.).

By thoroughly analysing the chemical profile of this new collection of *D. antarctica*, along with **1,** seven previously known diterpenes (**2**–**8**) have been found, occurring in varying concentrations in all individuals. In fact, they were also shown to possess interesting bioactivities previously (Table S1): deceptionin (**2**) is suggested to be related to a response to an environmental stressor [50]; the gracilane norditerpene **3** is an antioxidant with potential neuroprotective properties and could be related to chemical defence [51,55]; cadlinolide C (**4**) has been proposed to have anti-inflammatory activity [42]; the glaciolane norditerpene **5** has been suggested to have defensive activity [51]; membranolide (**6**) is a feeding deterrent against the amphipod *Gondogeneia antarctica* [68] and is active against MRSA [46]; aplysulphurin (**7**) is active against macrophages [46] and is cytotoxic for colon, lung, and carcinoma cancer cell lines [54]; and finally, tetrahydroaplysulphurin-1 (**8**) is related to protection of the cell membrane and activation of antioxidant pathways induced by oxidative stress [55]. Thus, *D. antarctica* is a relevant source of bioactive compounds. Whether the sponge itself or some symbionts within its microbiome are the producers of the natural compounds is an unsolved question that remains to be further studied.

### 3.2. Thermal Stress Experiment

The experimental design implemented here aimed to investigate the impact of exposure to temperatures up to 5 °C after 14 days on *D. antarctica*, according to IPCC 2022 predictions for the next century [69]. Along with minor rearranged spongiane, gracilane, and glaciolane derivatives (**1**, **3,** and **5**), the predominant diterpenes in *D. antarctica* were oxygenated metabolites with an aplysulphurane skeleton, such as DCP (**2**), MBN (**6**), APS (**7**), and TTS (**8**). In effect, aplysulphurane derivatives were the primary contributors to intra-group similarities and inter-group dissimilarities, indicating their relevance in defining the effect of stressors like aquarium conditions or temperature increase. Despite larger intra-group diversity in metabolite concentrations in the EC group, supporting previous findings that *D. antarctica* can exhibit varying chemical profiles even within the same ecosystem [49,50,70], the aquarium specimens showed a diverse molecular composition, with a trend to reduced intra-group variability, and increased the EC group’s dissimilarity from the AT and HS groups. Temperature seems to show a trend towards an increase in aplysulphurane derivatives, such as DCP (**2**), CLC (**4**), MBN (**6**), and TTS (**8**). Compounds like DDL (**1**), GRN (**3**), and GLN (**5**) were instead consistently present in all the specimens at the same level, indicating their constitutive function rather than an involvement in response to environmental pressures. In fact, their stable concentrations observed when moving from natural to aquarium conditions further suggest that certain routes may be less sensitive to external environmental changes, reinforcing the idea that not all metabolites respond equally to fluctuations in environmental pressures. On the other hand, the impact of the change from natural habitat to aquarium conditions was clearly appreciable on the level of two derivatives with an aromatic ring in their structure, i.e., MBN (**6**) and APS (**7**).

To date, detailed knowledge of the biosynthetic pathways leading to these diterpenes is still lacking. While we may hypothesise that key enzymes are more sensitive than others to environmental stressors including temperature changes, the final effect is an overall increment (30%, on average) in the total amount of the terpene pool in the specimens subjected to water temperature increase (3 mg total terpenes/g DW in the HS group vs. 2.3 mg/g in AT; Table S2). Conversely, a reduction in the level of main metabolites such as MBN (**6**) and APS (**7**) above all, as a response to changing environmental conditions (natural habitat *vs* aquarium), is somehow balanced by higher concentrations of other metabolites and particularly of major TTS (**8**) (Figure 3 and Table S2), thus keeping the total terpene level at 2.3 µg/mg DW vs. 2.6 µg/mg with a variation of about 10% (on average). This apparent stability could be indicative of a regulatory mechanism that ensures the homeostasis of these compounds towards specific stress events. Overall, the diverse tuning of the terpene pool in response to external stimuli highlights the complexity of the organisms’ adaptation mechanisms.

Further experiments and analyses are needed to confirm the observed trends. Increasing sample sizes and sampling areas is essential for more robust statistical analysis. This will help to determine which temperature increases cause significant changes in *D. antarctica*’s chemical profile, assessing its potential for chemical adaptation. High concentrations of compounds and wide variability in some metabolites might in fact reflect stress adaptations, metabolic activity changes, or responses to varying environmental conditions in aquarium control specimens and heat stress environments. Therefore, a deep investigation of the biosynthetic processes will contribute to defining a picture that is now only limited to a measure of a phenotypical character (metabolite content).

This study had been designed to establish whether there were chemical differences between a subtle temperature increase, tested before [50], from a slow temperature increase in *D. antarctica*. However, in the course of the experiment, all the specimens exposed to heat stress exhibited external morphological degradation at 4 °C, such as colour changes and necrotic spots, indicative of sponge health deterioration [71,72]. This deterioration agrees with the described changes inducing microbiome dysbiosis due to environmental stress [71,73,74,75,76,77]. To counteract this, natural products may help maintain the sponge’s inherent microbial community [78], which is crucial for its health and stability [79] and plays a significant role in the nitrogen and sulphur cycles within the holobiont [80,81]. However, in our experiment, the sponges had to be frozen because they were not doing well after 10 days. This is clearly a limitation of this study to be addressed in future experiments. Interestingly, it is quite possible that the sponge reacts to the temperature increase in different ways when a fast sudden change happens than when there is a slow gradual change [50], this study. This could remind us of the “boiling frog” apologue, with different responses when the water is boiling than when the temperature is gradually increasing. Although this is just a story, it may actually showcase the fact that sponges may react diversely in both cases by changing their chemical profile in different ways. Although on a different timescale, these changes could also support Pauly’s ecological theory of “shifting baselines”, since changes may occur slowly with a temperature increase but the “original” status is long gone [82]. This means that the sponge may have suffered slow environmental changes over time and thus we are now seeing dissimilar chemical profiles that are the result of accumulated impacts but are measured in comparison to baselines that have already changed. Further research is needed to ascertain this intriguing topic.

### 3.3. Further Remarks

Overall, the recorded chemical profiles are a fingerprint of *D. antarctica* individuals inhabiting Deception Island, an active stratovolcano complex with a submerged caldera at its centre (Port Foster). Many volcanic eruptions, fumarolic emissions, hot springs, and thermally altered soils have modified the landscape, creating environments markedly distinct from those in other regions of Antarctica. This phenomenon results in the waters being characterised by the presence of volcanoclastic particles in suspension [83,84] and chemical substances derived from local geothermal activity [85,86]. Additionally, fumarolic emissions and geothermal springs traversing the sedimentary seabed result in unusually high bottom water temperatures, ranging from 2 to 3 °C [87]. In Whalers Bay, temperatures higher than 2 °C have often been recorded [87]. This area, with some rocky spots, exhibits high species richness and unique shallow-water communities [88] that have recently been designated as a protected area based on our previous studies there (ASPA 145, subsite c; https://www.ats.aq/devph/en/apa-database/49 (accessed on 24 October 2024). The distinctive characteristic of this environment undoubtedly affects and stresses benthic filter feeders, such as *D. antarctica*, potentially contributing to the selection, evolution, and regulation of their natural compounds and microbiome, contributing to the variability in metabolite concentrations. This highlights the complexity of maintaining natural-like conditions in captivity. While our experiment was intended to closely mimic natural settings, unknown factors related to the aquarium environment led to diverse metabolic profiles. This sensitivity to the controlled environment may be linked to stress or other factors inherent to captivity. Therefore, we may expect that sponges exposed to stronger environmental pressures would exhibit more variable concentrations of natural compounds [89] and consequently the induction of cellular and physiological stress [90]. Furthermore, the natural variability in the different specimens of *D. antarctica*, as also highlighted in previous studies [49,50,70], should be taken into account when comparing results from different sample collections.

Discovering new metabolites could provide further insights into *D. antarctica*’s adaptation strategies, warranting continued investigation into specific factors influencing their metabolic profiles. Exploring how thermal stress and other environmental factors impact the health and long-term survival of these organisms is crucial. Investigating regulatory mechanisms of metabolite stability under stress conditions, including enzymatic activity and stress response pathways, could deepen our understanding of these organisms’ resilience and adaptability in their environment. This could also enhance management and conservation strategies for species under fluctuating environmental stresses, as mentioned above. Finally, the observed percentage of dissimilarity indicates the intricate nature of metabolic responses to environmental modifications and highlights the need of further exploration of the underlying biochemical mechanisms involved. Establishing the roles of specific enzymes, stress response proteins, and signalling pathways can provide deeper insights into the adaptation strategies of *D. antarctica* to such fluctuating environmental conditions. These insights could be crucial for predicting the long-term health and survival of these organisms amidst global change and could yield more effective conservation strategies in the future.

## 4. Materials and Methods

### 4.1. Collection and Sample Preservation of D. antarctica

**Figure 4:**
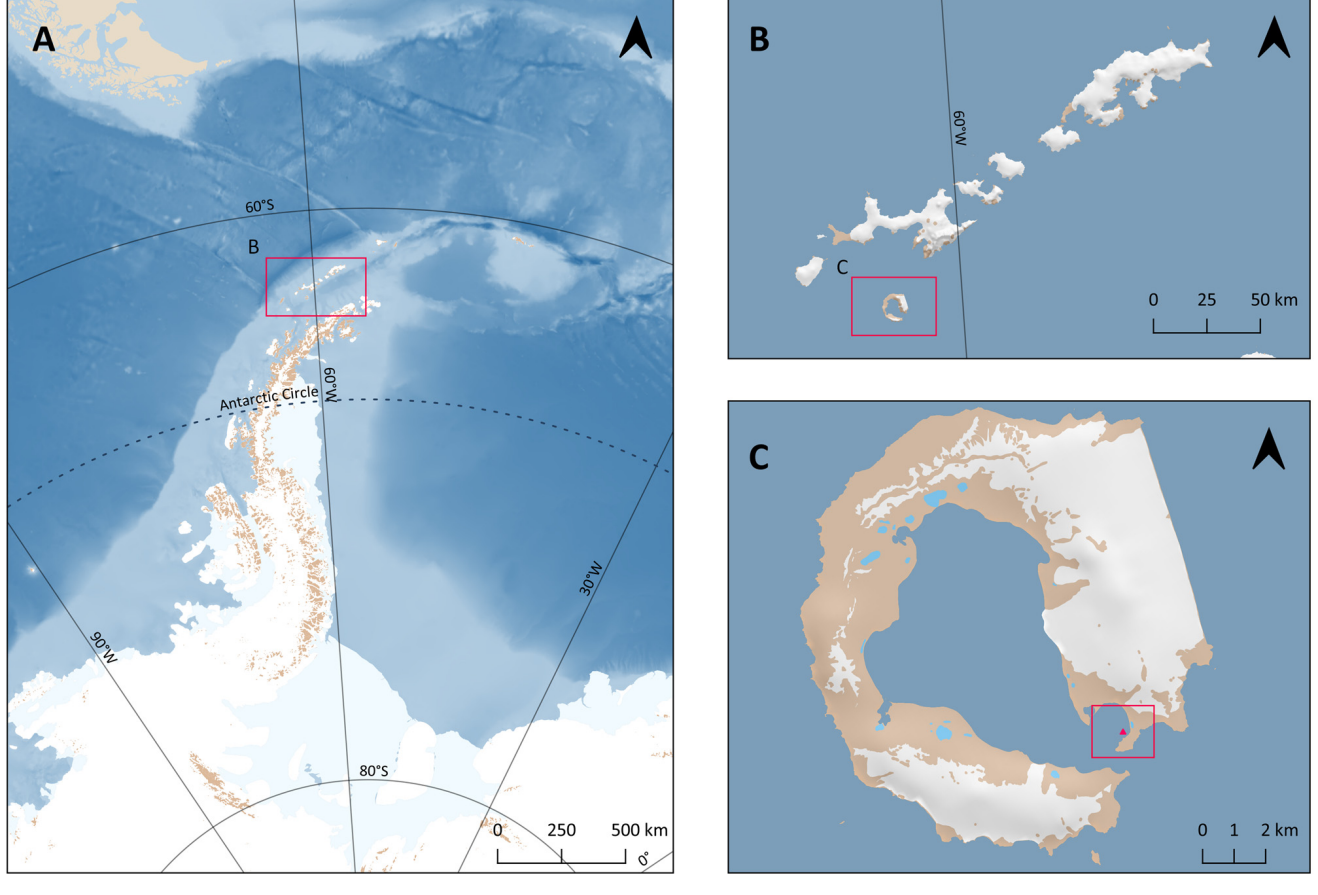
Maps of the sampling area: (**A**) an overview of the WAP and South Shetland Islands (SSIs), (**B**) a close-up of the SSIs, and (**C**) a detailed map of Deception Island and the specific sampling area (Cormoran Town, Whalers Bay).

Fifteen specimens of *D. antarctica* were collected by SCUBA diving from the research vessel BIO Hespérides in Cormoran Town, Whalers Bay, Deception Island (South Shetland Islands, Antarctica; 62°59.210′ S, 60°33.228′ W) (Figure 4) during the austral summer period of December 2022 to March 2023. Cormoran Town, within Whalers Bay, is a shallow rocky-bottom area (20–25 m depth) covered by a rich marine invertebrate community of sponges, tunicates, soft corals, bryozoans, etc. The identification of the sponge in the laboratory was based on the current World Porifera Database [91].

Five specimens were immediately frozen after collection and stored at −20 °C to be used as the environmental temperature control (EC). Simultaneously, the remaining ten specimens were placed in seawater aquaria at 0 °C for the heat stress experiment: five to establish an aquarium control (AT) and five for the heat stress treatment (HS).

### 4.2. Heat Stress Experiment

The experimental design comprised a one-week quarantine phase, followed by 14 days of exposure to the two different temperature regimes. The maximum temperature chosen for the experiment was based on projections from the Intergovernmental Panel on Climate Change for the year 2022, considering the average water temperature and anticipated changes by 2050 and 2100 [69]. To investigate the impact of heat stress on the sponge natural products, ten individuals of *D. antarctica* were placed in aquaria and subjected to two distinct temperatures. The experiment included (1) five individuals as a control temperature (AT) of local seawater at a constant 0 °C ± 0.2 °C and (2) five individuals as a heat stress temperature (HS), consisting of a gradual increase from 0 °C to 5 °C ± 0.3 °C (ΔT = 1 °C/2.5 days) for two weeks. However, when the temperature reached 4 °C, after ten days, the experiment was finished due to the low viability of the samples. The temperature measurements and controls were obtained by using two types of thermometers: a mercury thermometer and a digital controller (Aqua Medic T controller twin) connected to heating and/or cooling units (Titan 150, Aqua Medic, Bissendorf, Germany). All the specimens were allocated into compartmentalised tanks (125 L). Seawater circulated through all the compartments and aeration was provided to each tank. At the end of the experimental period, all the individuals were frozen and stored at −20 °C. Later, the frozen specimens were transported at −20 °C to the Istituto di Chimica Biomolecolare (ICB-CNR, Pozzuoli, Italy). Once there, all the samples were lyophilised and stored at −20 °C until further processing in the chemical laboratory.

### 4.3. General Procedures

All the chemicals were of analytical reagent grade and all the solvents were of HPLC/LC-MS grade (>99% purity) from Merck (Milan, Italy), used without further purification unless otherwise specified. The TLC plates (Silicagel 60 F_254_) and silica gel powder (Silicagel 60, 0.063–0.200 mm) were sourced from Merck (Milan, Italy). The GC-MS analyses were performed utilising an ion-trap MS instrument in electron ionisation mode (70 eV) (Polaris Q, Thermo Scientific, Milan, Italy) integrated with a GC system (GCQ, Thermo Scientific). A 5% phenyl/methyl polysiloxane column (VF-5ms, 30 m × 0.25 mm × 0.25 μm; Agilent Technologies, Cernusco sul Naviglio (MI), Italy) was employed with helium as the carrier gas. The HPLC analyses and fractionation were carried out using a high-performance liquid chromatography system equipped with binary LC-20AD pumps and a Diode Array Detector SPD-M20A (Shimadzu, Milan, Italy).

The optical rotation was measured on a P-2000 digital polarimeter at 589 nm (Jasco, Milan, Italy). The FT-IR spectrum was recorded on a FT/IR 4100 spectrophotometer (Jasco, Milan, Italy). The UV spectrum was acquired on a V-650 Spectrophotometer (Jasco, Milan, Italy). The one-dimensional and two-dimensional NMR spectra were recorded on a Bruker AVANCE™ III HD-400, equipped with a CryoProbe™ Prodigy, or on a Bruker DRX-600 equipped with a TXI CryoProbe™ (Bruker, Milan, Italy) in CDCl_3_ (δ_H_ values reported refer to residual solvent proton at 7.26; δ_C_ values refer to solvent carbon at 77.0 ppm). The high-resolution mass spectra were acquired on a Q-Exactive Hybrid Quadrupole-Orbitrap Mass Spectrometer (Thermo Scientific, Milan, Italy).

### 4.4. Natural Product Extraction and Metabolomic Analysis

To obtain the chemical profile of *D. antarctica*, ca. 100 mg of each freeze-dried specimen was extracted with dichloromethane (DCM) (3 × 10 mL). Phytyl acetate (80 µg) was added as an internal standard (IS) for quantitative analysis. The combined extracts were concentrated under a N_2_ stream and re-dissolved in 500 μL *tert*-butyl methyl ether (MTBE) for the GC-MS analysis. The following temperature program was applied: an initial 160 °C was held for 3 min, then increased 5 °C min^−1^ up to 210 °C, and followed by 2 °C min^−1^ up to 260 °C. Subsequently, the temperature was increased at a rate of 15 °C min^−1^ to 310 °C and held for 3 min. The split flow was set to 10 mL min.^1^ with a transfer line temperature of 280 °C, an inlet temperature of 290 °C, and an ion source temperature of 250 °C. Full-scan mode was employed with a mass range of 40–500 *m*/*z*. A 2 μL aliquot was injected for each analytical run and all the samples were analysed in duplicate.

### 4.5. Isolation and Characterisation of Terpenes

The freeze-dried specimens of *D. antarctica* (10 g) were extracted with DCM (3 × 200 mL); the raw extract (264 mg) was fractionated by silica gel chromatography on a column by using a gradient elution of diethyl ether (EE) in petroleum ether (PE): a total of 18 fractions were collected and analysed by TLC, ^1^H-NMR, and GC-MS. A comparison with our previous data and other literature reports [47,50] allowed for the straightforward identification of diterpenes **2**–**8** in fractions eluted by PE/EE from 9:1 to 1:1. Compound **1** was detected in subfractions eluted by PE/EE 9:1 and was obtained in a pure form following a further fractionation step by semipreparative normal phase–high-performance liquid chromatography (NP-HPLC) on a Kromasil KR100-5-Sil column (Merck, 250 × 10 mm, 5 µm) at a flow rate of 3.5 mL min^−1^ with PDA detector monitoring at 220, 260, and 272 nm; for the elution, *n*-hexane (A) and *n*-hexane/isopropanol 97:3 (B) were used with 60:40 isocratic conditions over a 30 min run time (DDL, **1**, 1.3 mg, and RT 12.5 min). Cadlinolide C (**4**, 0.8 mg, and RT 19.0 min) was isolated along with **1** from the same silica subfractions. The remaining diterpenes were purified by HPLC as previously reported [50].

### 4.6. Dendrillolactone (**1**)

Colourless oil. HRESI^+^-MS = 383.2199 *m*/*z* on [M+Na]^+^ accounting for C_22_H_32_O_4_ (theor 383.2193). IR (film) ν_max_ = 1830, 1735, 1626, 1565, 1456, 1371, and 1245 cm^−1^; UV λ_max_ (ε) = 220 nm (1074); and [α]_D_ (c = 0.0067, CHCl_3_) = +70.0. For the NMR data, see Table 1.

### 4.7. Quantitative Molecular Assessment and Statistical Insights

Quantitative processing of the raw data was performed by using Thermo Xcalibur software (Thermo Fisher Scientific Inc., version 2.2 SP1.48, Waltham, MA, USA). For each metabolite, the amount was calculated by peak area normalisation to the IS area and expressed as μg/mg sponge in dry weight (DW). From the data obtained, the means and standard deviations for each group were calculated and represented in a barplot. The dataset for each sample was imported into PRIMER-E (Primer 6 version 6.1.16 and Permanova+ version 1.0.6) following the literature recommendations [92]. The chemical data for all the samples were transformed using the fourth root to achieve homoscedasticity, and a Bray–Curtis similarity matrix was created for the eight principal compounds. A permutational analysis of variance (PERMANOVA) was performed for the three groups of samples (EC, AT, and HS). Wilcoxon pairwise tests were used for comparisons between the control groups and the treatment group. To discriminate the variability in the molecules according to the effect of heat stress, a similarity analysis (SIMPER) was conducted with 999 permutations, using the treatment groups as factors.

## 5. Conclusions

The present study provides an analysis of the variability in the chemical profile of *D. antarctica* under gradual thermal stress. The results show a trend towards chemical changes in the sponge’s natural products related to the increase in seawater temperatures. Furthermore, following a metabolomics approach, a novel diterpene derivative here named dendrillolactone (**1**), with a rare rearranged spongiane β-lactone skeleton, has been identified, isolated, and chemically characterised from this sponge collection. Overall, our findings highlight the critical need for further research on the chemical compounds of *D. antarctica* and their functional roles under environmental stress. Understanding these mechanisms is crucial for predicting the resilience and future of Antarctic benthic ecosystems in the face of global change. Further studies could also explore the potential biotechnological applications of these metabolites in stress resilience.

## Figures and Tables

**Table 1:** ^1^H and ^13^C NMR data of dendrillolactone (**1**) (CDCl_3_, 600 MHz).

Position	^1^H, δ, Mult, *J* (Hz)	^13^C, ppm
1	1.00, m 1.93, m	38.3
2	1.49, m	21.3
3	1.12, m 1.28, m	39.6
4	-	31.4
5	1.03, d, 14.2 1.94, d, 14.2	49.9
6	3.93, ddd, 10.9, 6.1, 6.1	54.3
7	2.67, m 3.07, dd, 16.0, 10.9	30.4
8	-	133.3
9	-	138.5
10	-	39.6
11	2.04, m 2.31, m	26.1
12	1.30, m 1.85, m	26.7
13	1.71, m	36.3
14	2.44, m	50.2
15	4.74, dd, 6.1, 3.7	80.1
16	4.06, dd, 11.2, 6.0 4.16, dd, 11.2, 6.7	68.1
17	0.80, s	26.2
18	0.87, s	33.1
19	0.99, s	30.8
20	-	171.9
O*CO*CH_3_		170.8
OCO*CH_3_*	2.08, s	20.9

**Table 2:** SIMPER analysis for the detection of the metabolite contribution to the dissimilarity between treatments. Factors include the following: EC—environmental control; AT—aquarium temperature control; and HS—heat stress treatment. DDL—dendrillolactone (**1**); DCP—deceptionin (**2**); GRN—the gracilane norditerpene **3**; CLC—cadlinolide C (**4**); GLN—the glaciolane norditerpene **5**; MBN—membranolide (**6**); APS—aplysulphurin (**7**); and TTS—tetrahydroaplysulphurin-1 (**8**). The values calculated by SIMPER are as follows: average dissimilarity (groups), average abundance (I and II), average dissimilarity (metabolite), average dissimilarity/standard deviation (SD), contribution percentage (%), and cumulative percentage (%). In bold, the molecules contributing the most.

Factors	Average Dissimilarity (Groups)	Metabolite	Average Abundance (I)	Average Abundance (II)	Average Dissimilarity (Metabolite)	Dissimilarity/SD	Contribution (%)	Cumulative (%)
EC (I) and AT (II)	10.91	**MBN**	**0.80**	**0.72**	**2.44**	**1.57**	**22.34**	**22.34**
		**TTS**	**0.86**	**0.84**	**1.98**	**1.22**	**18.15**	**40.49**
		**APS**	**0.70**	**0.56**	**1.94**	**1.27**	**17.75**	**58.23**
		CLC	0.57	0.65	1.02	1.31	9.31	67.54
		DCP	0.88	0.85	0.96	1.47	8.81	76.35
		DDL	0.59	0.63	0.90	1.41	8.22	84.56
		GLN	0.57	0.52	0.85	1.50	7.77	92.33
		GRN	0.48	0.51	0.84	1.49	7.67	100.00
EC (I) and HS (II)	11.27	**MBN**	**0.80**	**0.80**	**2.36**	**1.75**	**20.93**	**20.93**
		**APS**	**0.70**	**0.58**	**1.81**	**1.54**	**16.05**	**36.98**
		**TTS**	**0.86**	**0.97**	**1.80**	**2.10**	**15.95**	**52.93**
		CLC	0.57	0.69	1.42	1.89	12.60	65.54
		DCP	0.88	0.90	1.38	1.39	12.21	77.74
		GLN	0.57	0.50	0.88	1.12	7.85	85.59
		DDL	0.59	0.60	0.84	1.41	7.47	93.06
		GRN	0.48	0.52	0.78	1.25	6.94	100.00
AT (I) and HS (II)	10.80	**TTS**	**0.84**	**0.97**	**2.60**	**1.37**	**24.06**	**24.06**
		**MBN**	**0.72**	**0.80**	**1.60**	**1.44**	**14.80**	**38.87**
		**DCP**	**0.85**	**0.90**	**1.54**	**1.50**	**14.25**	**53.12**
		CLC	0.65	0.69	1.30	1.57	12.08	65.20
		APS	0.56	0.58	1.26	1.50	11.65	76.85
		GLN	0.52	0.50	0.93	1.49	8.64	85.49
		DDL	0.63	0.60	0.80	1.41	7.37	92.86
		GRN	0.51	0.52	0.77	1.54	7.14	100.00

**Table 3:** Similarity percentage (SIMPER) analysis used to assess the contribution of each metabolite recorded in *D. antarctica.* EC—environmental control; AT—aquarium temperature control; and HS—heat stress treatment. DDL—dendrillolactone (**1**); DCP—deceptionin (**2**); GRN—the gracilane norditerpene **3**; CLC—cadlinolide C (**4**); GLN—the glaciolane norditerpene **5**; MBN—membranolide (**6**); APS—aplysulphurin (**7**); and TTS—tetrahydroaplysulphurin-1 (**8**). The values calculated by SIMPER are as follows: average similarity (groups), average abundance, average similarity (metabolite), average similarity/standard deviation (SD), contribution percentage (%), and cumulative percentage (%). In bold, the molecules contributing the most.

Factors	Average Similarity (Groups)	Metabolite	Average Abundance	Average Similarity (Metabolite)	Similarity/SD	Contribution (%)	Cumulative (%)
EC	89.53	**DCP**	**0.88**	**15.29**	**15.96**	**17.08**	**17.08**
		**TTS**	**0.86**	**15.25**	**6.39**	**17.03**	**34.12**
		**MBN**	**0.80**	**11.18**	**3.21**	**12.48**	**46.60**
		APS	0.70	10.66	4.57	11.90	58.50
		GLN	0.57	9.78	20.65	10.92	69.42
		DDL	0.59	9.75	11.47	10.89	80.31
		CLC	0.57	9.75	24.92	10.89	91.20
		GRN	0.48	7.88	11.44	8.80	100.00
AT	88.77	**TTS**	**0.97**	**15.07**	**7.02**	**16.98**	**16.98**
		**DCP**	**0.90**	**14.13**	**7.52**	**15.92**	**32.89**
		**MBN**	**0.80**	**12.83**	**9.85**	**14.45**	**47.34**
		CLC	0.69	10.94	7.30	12.32	59.66
		DDL	0.60	9.98	16.97	11.24	70.90
		APS	0.58	9.30	8.08	10.47	81.38
		GRN	0.52	8.62	14.50	9.71	91.09
		GLN	0.50	7.91	9.31	8.91	100.00
HS	87.61	**DCP**	**0.85**	**14.9**	**13.86**	**17.00**	**17.00**
		**TTS**	**0.84**	**12.47**	**3.42**	**14.23**	**31.24**
		**MBN**	**0.72**	**11.85**	**6.72**	**13.52**	**44.76**
		DDL	0.63	11.06	14.71	12.63	57.39
		CLC	0.65	10.95	12.80	12.50	69.89
		GLN	0.52	8.83	12.27	10.08	79.97
		APS	0.56	8.82	5.20	10.06	90.03
		GRN	0.51	8.73	12.89	9.97	100.00

## Data Availability

The data presented in this study are available upon request from the corresponding author.

## Supplementary Materials

The following supporting information can be downloaded at the following: https://www.mdpi.com/article/10.3390/md23010010/s1. Table S1. Natural products isolated and characterised from *Dendrilla antarctica* with their described bioactivities; Table S2. Concentrations of the terpene derivatives in *D. antarctica* specimens analysed in this study; Figure S1. EIMS spectrum of dendrillolactone (DDL; **1**); Figure S2. EIMS spectrum of deceptionin (DCP; **2**); Figure S3. EIMS spectrum of the gracilane norditerpene **3** (GRN); Figure S4. EIMS spectrum of cadlinolide C (CLC; **4**); Figure S5. EIMS spectrum of the glaciolane norditerpene **5** (GLN); Figure S6. EIMS spectrum of membranolide (MBN; **6**); Figure S7. EIMS spectrum of aplysulphurin (APS; **7**); Figure S8. EIMS spectrum of tetrahydroaplysulphurin-1 (TTS; **8**); Figure S9. HRESI^+^-MS spectrum of the [M+Na]^+^ adduct of dendrillolactone (**1**); Figure S10. ^1^H NMR spectrum of dendrillolactone (**1**, CDCl_3_, 600 MHz); Figure S11. ^13^C NMR spectrum of dendrillolactone (**1**, CDCl_3_, 150 MHz); Figure S12. ^1^H, ^1^H COSY NMR spectrum of dendrillolactone (**1**, CDCl_3_, 600 MHz); Figure S13. ^1^H, ^1^H TOCSY NMR spectrum of dendrillolactone (**1**, CDCl_3_, 600 MHz); Figure S14. ^1^H, ^13^C edited-HSQC NMR spectrum of dendrillolactone (**1**, CDCl_3_, 400 MHz); Figure S15. ^1^H, ^13^C HMBC NMR spectrum of dendrillolactone (**1**, CDCl_3_, 600 MHz).
