# Supplementary material for: Chemical Changes Under Heat Stress and Identification of Dendrillolactone, a New Diterpene Derivative with a Rare Rearranged Spongiane Skeleton from the Antarctic Marine Sponge Dendrilla antarctica
Source: Mar Drugs. 2024 Dec 28;23(1):10. doi: 10.3390/md23010010 (PMC11767012; doi:10.3390/md23010010)

Supplementary Material

Table S1. Natural products isolated and characterised from *Dendrilla antarctica* with their described bioactivities.

Table S2. Concentrations of the terpene derivatives in *D. antarctica* specimens analysed in this study.

Figure S1. EIMS spectrum of dendrillolactone (DDL; **1**).

Figure S2. EIMS spectrum of deceptionin (DCP; **2**).

Figure S3. EIMS spectrum of the gracilane norditerpene **3** (GRN).

Figure S4. EIMS spectrum of cadlinolide C (CLC; **4**).

Figure S5. EIMS spectrum of the glaciolane norditerpene **5** (GLN).

Figure S6. EIMS spectrum of membranolide (MBN; **6**).

Figure S7. EIMS spectrum of aplysulphurin (APS; **7**).

Figure S8. EIMS spectrum of tetrahydroaplysulphurin-1 (TTS; **8**).

Figure S9. HRESI$^+$-MS spectrum of the $[M+Na]^+$ adduct of dendrillolactone (**1**).

Figure S10. ^1H NMR spectrum of dendrillolactone (**1**, $CDCl_3$, 600 MHz).

Figure S11. ^{13}C NMR spectrum of dendrillolactone (**1**, $CDCl_3$, 150 MHz).

Figure S12. ^1H,^1H COSY NMR spectrum of dendrillolactone (**1**, $CDCl_3$, 600 MHz).

Figure S13. ^1H,^1H TOCSY NMR spectrum of dendrillolactone (**1**, $CDCl_3$, 600 MHz).

Figure S14. ^1H,^{13}C edited-HSQC NMR spectrum of dendrillolactone (1, $CDCl_3$, 400 MHz).

Figure S15. ^1H,^{13}C HMBC NMR spectrum of dendrillolactone (**1**, $CDCl_3$, 600 MHz).

Table S1. Natural products isolated and characterised from *Dendrilla antarctica* with their described bioactivities.

Natural products	Location	Bioactivity	References
Aplysulphurin	Ross Island and Anvers Island (Antarctica)	Extracts of *Goniobranchus reticulatus* and the sponge *Darwinella tango* tested cytotoxic to colon (SW620) (IC50 10.5 μM), lung (NCIH460) (IC50 13.7 μM), and cervical carcinoma (KB3-1) (IC50 9.8 μM) cancer cell lines*; inhibits *Leishmania donovani* (IC50 3.1 μM), *Plasmodium falciparum* (100% at ≤13 μM), and the formation of *Staphylococcus aureus* (MRSA) biofilm (29.4% at 50 μg/ml and 6.5% at 25 μg/ml)	[36,40,46,53*,54*]
Cadlinolide C	Anvers Island (Antarctica)	Related to anti-inflammatory activity and inhibition of MRSA biofilm formation (90% at 100 μg/ml and 50% at 50 μg/ml)	[42,46, 47*]
Darwinolide	Anvers Island (Antarctica)	Demonstrates a 4-fold selectivity against the biofilm of MRSA (MIC 132.9 μM), with an IC50 of 33.2 μM against the biofilm and 73.4 μM in J774 macrophage cells	[44]
Deceptionin	Deception Island (Antarctica)	Potential relationship with a response to environmental stressors	[50]
Dendrillin A	Anvers Island (Antarctica)	Active against *L. donovani* (IC50 6.0 μM)	[38, 46]
Dendrillin B	Anvers Island (Antarctica)	Active against *L. donovani* (IC50 3.5 μM) and MRSA biofilm (90.7% at 100 μg/ml)	[46]
Dendrillin C	Anvers Island (Antarctica)	Active against MRSA biofilm (50% at 25 μg/ml)	[46]
Dendrillin D	Anvers Island (Antarctica)	Activity not described	[46]
Dendrinolide	Terranova Bay (New Zealand)	Possible potential defensive activity	[39]
Glaciolide	Anvers Island (Antarctica)	Active against *L. donovani* (IC50 8.8 μM)	[46]
Glaciolane norditerpene	Ross Island (King George)	Possible association with defensive activity	[41,51, 52*]
Glacionolide	Terranova Bay (New Zealand)	Activity not described	[39]
Gracilin A	Ross Island and King George (Antarctica), and repository source	Antioxidant activity and neuroprotective described in *Spongionella sp.* (concentration of 0.1 μM and 1 μM)*	[36,41,55*]
Gracilane norditerpene	King George (Antarctica) and repository source	Potential antioxidant activity with probable neuroprotective properties*	[41,55*]
Membranolide	Ross Island and Anvers Island (Antarctica)	Active against *Bacillus subtilis* (100 μg/disk); feeding deterrent and active against MRSA (ca. 100% at 25 μg/ml) and active against *L. donovani* (IC50 9.7 μM)	[36,37,40,46,49]

Membranolides B (renamed Membranoid A)	Anvers Island (Antarctica)	No significant activity against *L. donovani* (IC50 > 29 μM) and low toxicity in mammalian J774A.1 cells (IC50 of 54.6 μM)	[40,48]
Membranolide C (renamed Membranoid C)	Anvers Island (Antarctica)	Antibiotic activity against *E. coli* (8 mm, 200 μg/disk), antifungal activity against *C. albicans* (4 mm, 200 μg/disk), active against *L. donovani* (IC50 6.5 μM) and no significant toxicity in mammalian J774A.1 cells (IC50 > 133 μM)	[40,48]
Membranolide D (renamed Membranoid E)	Anvers Island, Antarctica	Antibiotic activity against *S. aureus* (7 mm) and *E. coli* (6 mm, 200 μg/disk); antifungal activity against *C. albicans* (9 mm, 200 μg/disk), *L. donovani* (IC50 6.6 μM) and low toxicity in mammalian J774A.1 cells (IC50 95.0 μM)	[40,48]
Membranoid B, D, F, H	Anvers Island (Antarctica)	Active against *L. donovani*: B (IC50 0.8 μM), D (IC50 1.4 μM), F (IC50 26.7 μM) and H (IC50 12.0 μM); low toxicity in mammalian J774A.1 cells (IC50 > 133 μM)	[48]
Membranoid G	Anvers Island (Antarctica)	Active against *L. donovani* (IC50 1.9 μM); inhibition of htop1 starting at 150 μM, maximal at 200 μM in pre-incubation, from 80 μM; cytotoxicity in cancer cell lines at 100 μM, with EC50 ranging from 0.007 mM to 1.5 mM depending on the cell line	[48,57]
Tetrahydroaplysulphurin-1	Anvers Island (Antarctica)	Active against *L. donovani* (IC50 3.5 μM; reduces MRSA biofilm formation (31.1% inhibition at 50 μg/ml); cell membrane protection and activation of antioxidant pathways induced by oxidative stress (concentration of 0.1 μM and 1 μM, co-incubated with 200 μM hydrogen peroxide (H_2O_2))*	[40,46,48,55*]
4,5,8-trihydroxyquinoline-2-carboxylic acid	Ross Island (Antarctica)	Antibacterial activity against *Staphylococcus aureus*, *Vibrio anguillarum*, and *Beneckea harveyi* B-392 at a dose of 100 μg/disk	[56]
9,11-dihydrogracilin A	Ross Island (Antarctica), Tierra del Fuego (Argentina) and Terranova Bay (New Zealand)	Active against *Bacillus subtilis* (100 μg/disk); immuno-modulatory and anti-inflammatory activity on peripheral blood mononuclear cells (PBMC) and human keratinocytes (HaCaT) (0.3-10 μM in vitro and 1 μmol/cm^2 in vivo); antifouling properties (100 mg); effective against *L. donovani* (IC50 9.1 μM), ; reduces MRSA biofilm formation (29.1% at 50 μg/ml); cytotoxicity in macrophages J774A.1 (IC50 23 μM); activity against *P. falciparum* (0% inhibition at ≤16 μM)	[30,36,39,43–46]
9,11-dihydrogracillinone A	Tierra del Fuego (Argentina)	Antifouling activity (25 mg%)	[30]

*These bioactivities have been previously reported in other species; however, the corresponding molecules are also present in *Dendrilla antarctica*.

Table S2. Concentrations of the terpene derivatives in *D. antarctica* specimens analysed in this study. Values are expressed as mean µg/mg of dry sponge weight (DW) ± SD (n = 5). EC = environmental control, AT = aquarium temperature control, and HS= heat stress treatment. DDL – dendrillolactone (**1**); DCP – deceptionin (**2**); GRN – the gracilane norditerpene **3**; CLC – cadlinolide C (**4**); GLN – the glaciolane norditerpene **5**; MBN – membranolide (**6**); APS – aplysulphurin (**7**); TTS– tetrahydroaplysulphurin-1 (**8**).

GROUP	DDL	DCP	GRN	CLC	GLN	MBN	APS	TTS	Total amount (average)
**EC**	0,14 ± 0.08	0,62 ± 0.25	0,06 ± 0.04	0,11 ± 0.05	0,11 ± 0.05	0,64 ± 0.54	0,37 ± 0.14	0,57 ± 0.20	2,62
**AT**	0,17 ± 0.08	0,56 ± 0.27	0,08 ± 0.05	0,20 ± 0.14	0,09 ± 0.06	0,34 ± 0.26	0,13 ± 0.11	0,76 ± 0.33	2,33
**HS**	0,14 ± 0.06	0,77 ± 0.41	0,08 ± 0.04	0,27 ± 0.15	0,07 ± 0.04	0,49 ± 0.38	0,13 ± 0.11	1,05 ± 0.52	3,00

Figure S1. EIMS spectrum of dendrillolactone (DDL; **1**).

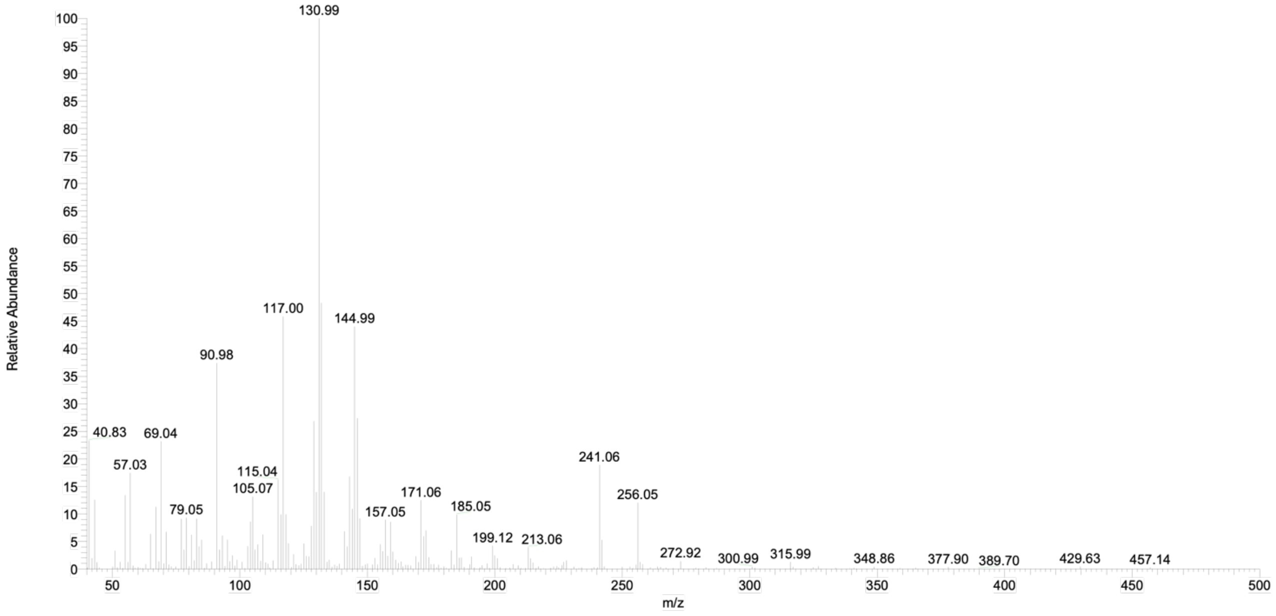

Figure S2. EIMS spectrum of deceptionin (DCP; **2**).

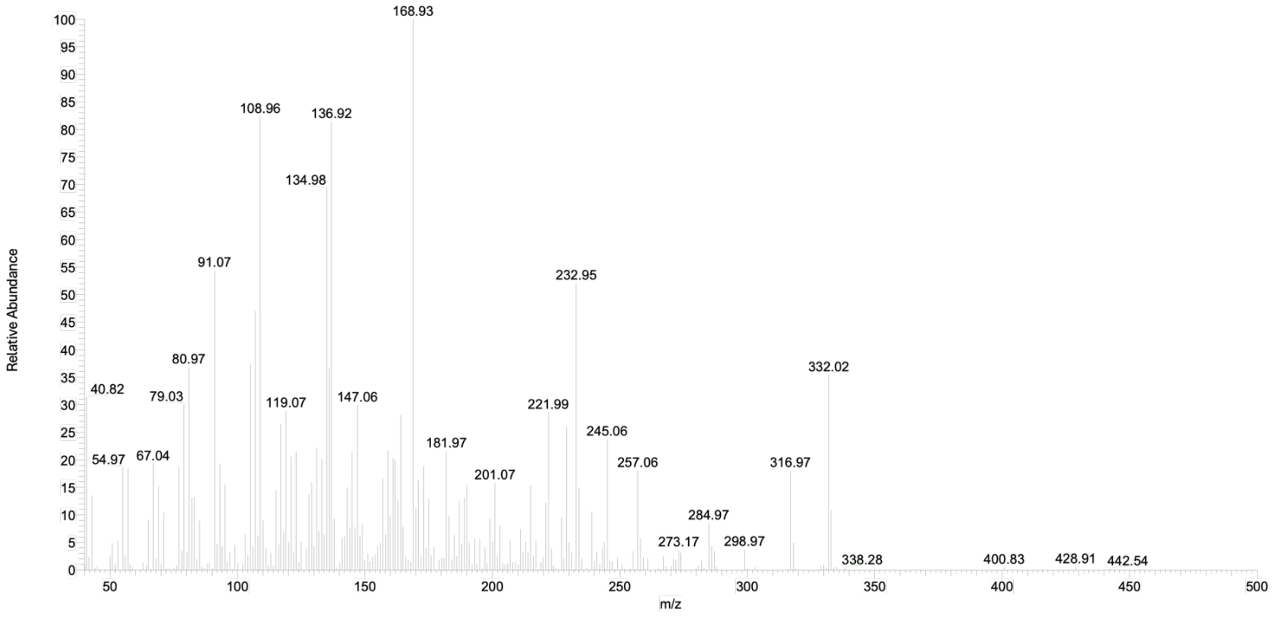

Figure S3. EIMS spectrum of the gracilane norditerpene **3** (GRN).

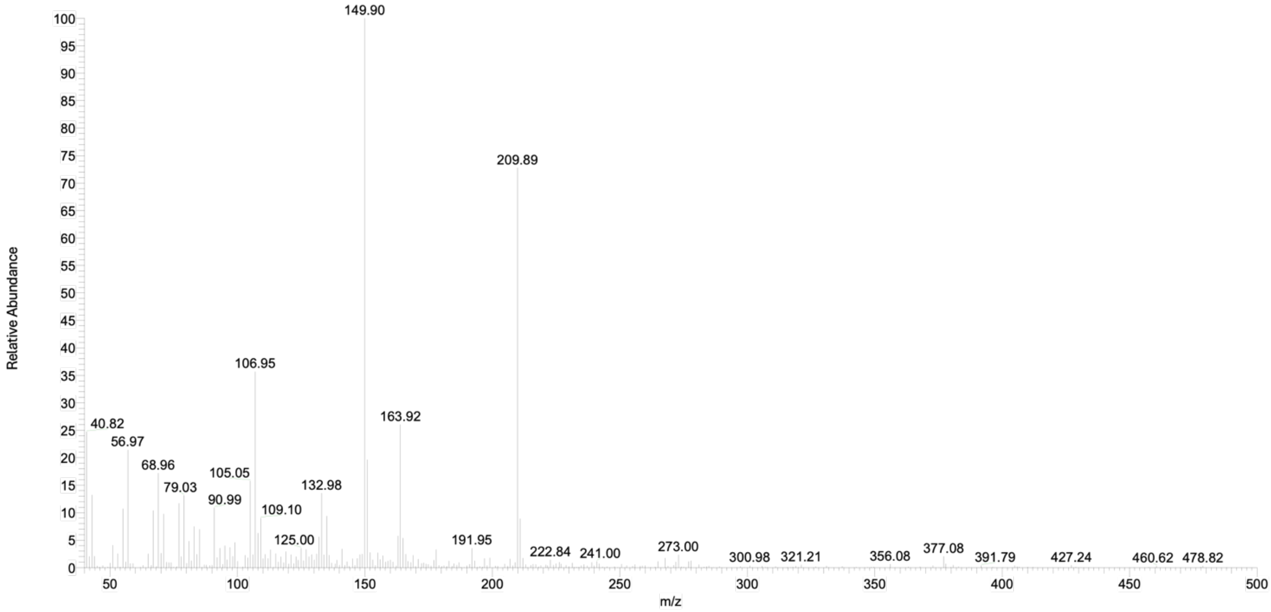

Figure S4. EIMS spectrum of cadlinolide C (CLC; **4**).

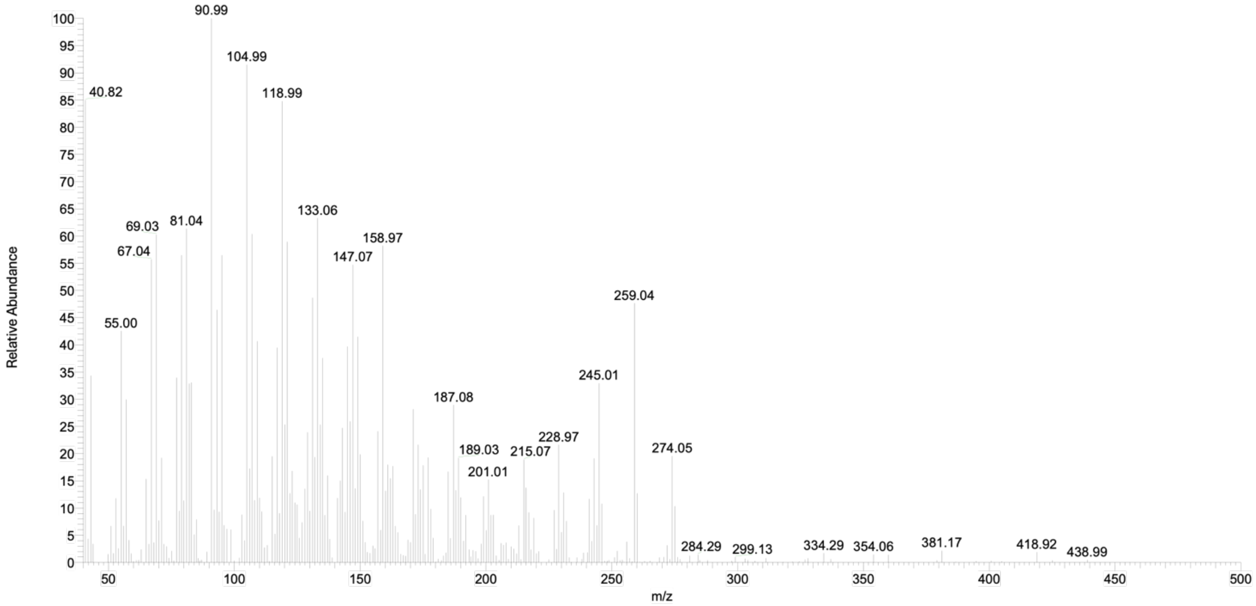

Figure S5. EIMS spectrum of the glaciolane norditerpene **5** (GLN).

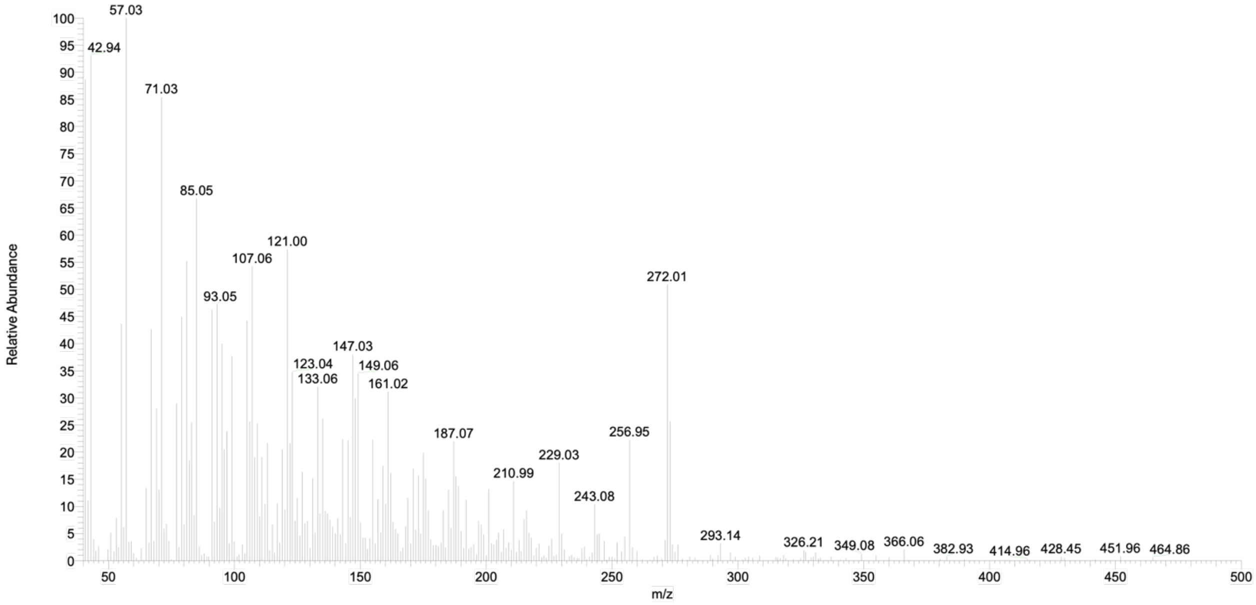

Figure S6. EIMS spectrum of membranolide (MBN; **6**).

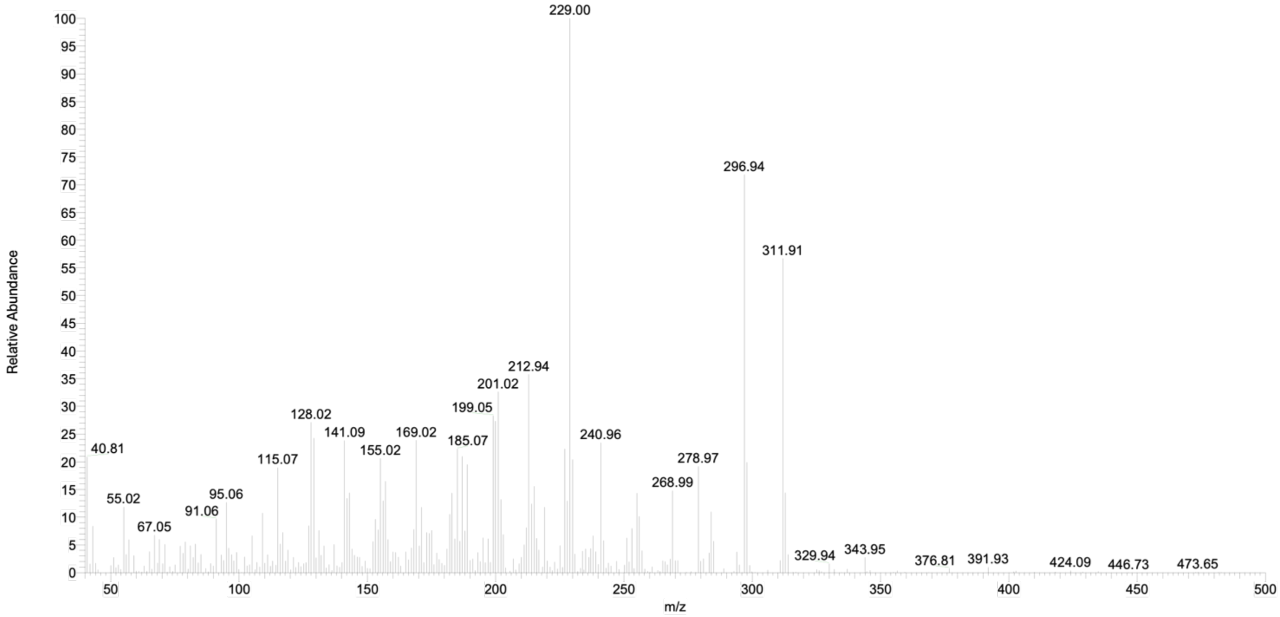

Figure S7. EIMS spectrum of aplysulphurin (APS; **7**).

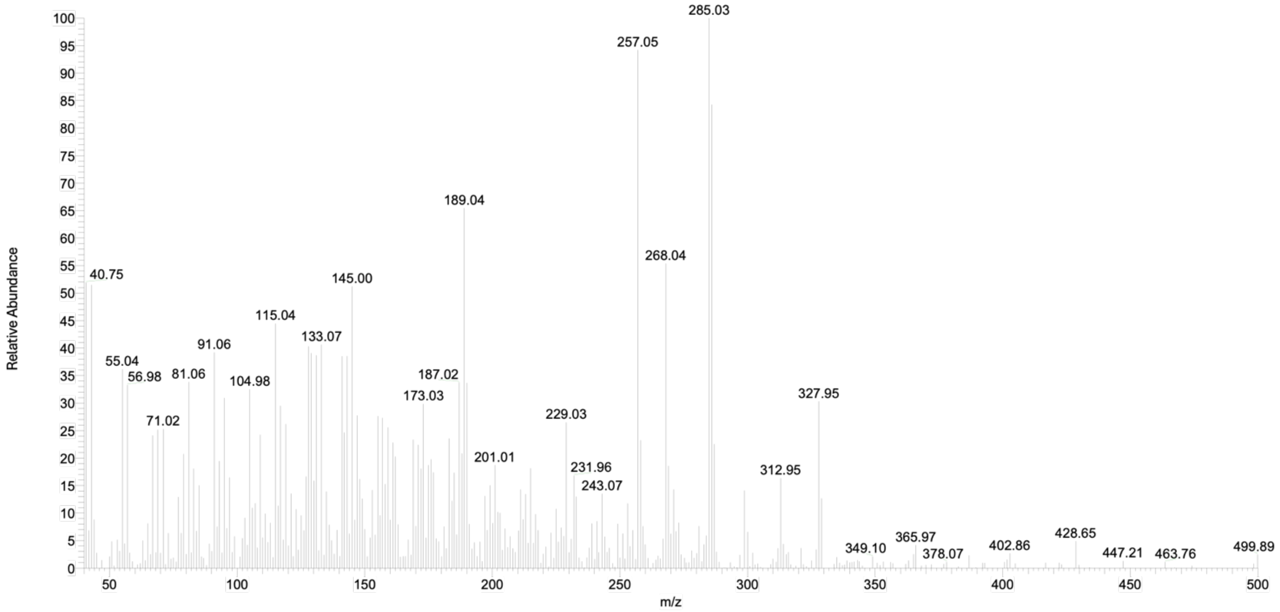

Figure S8. EIMS spectrum of tetrahydroaplysulphurin-1 (TTS; **8**).

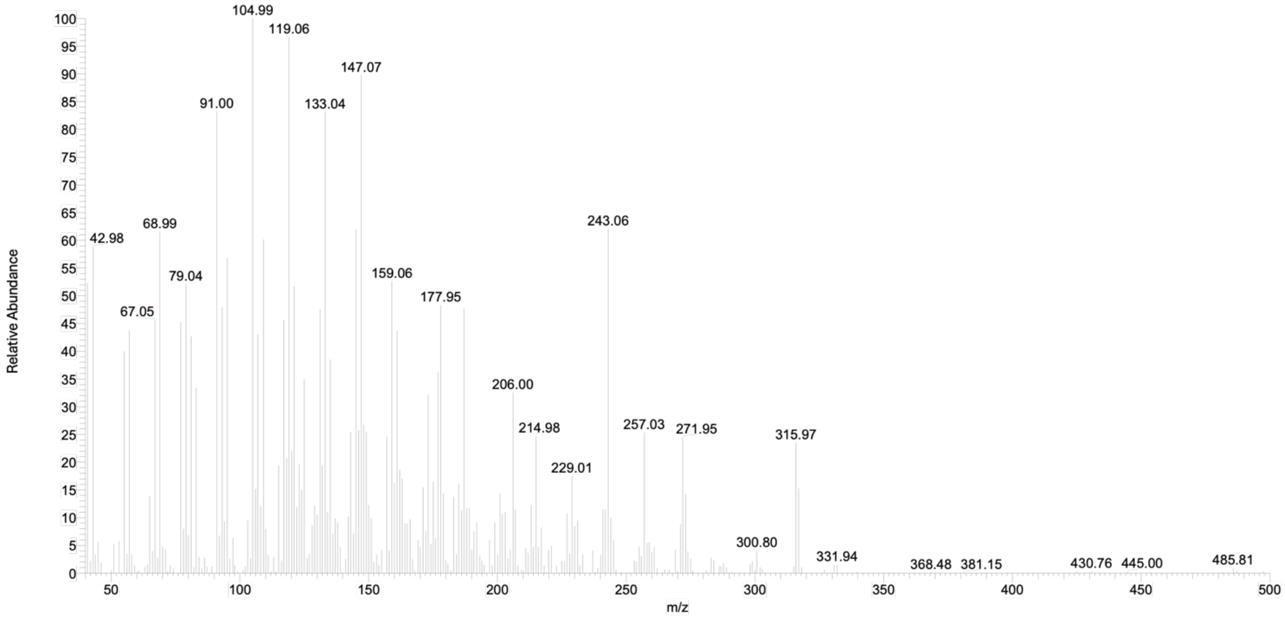

Figure S9. HRESI$^+$-MS spectrum of the $[M+Na]^+$ adduct of dendrillolactone (**1**)

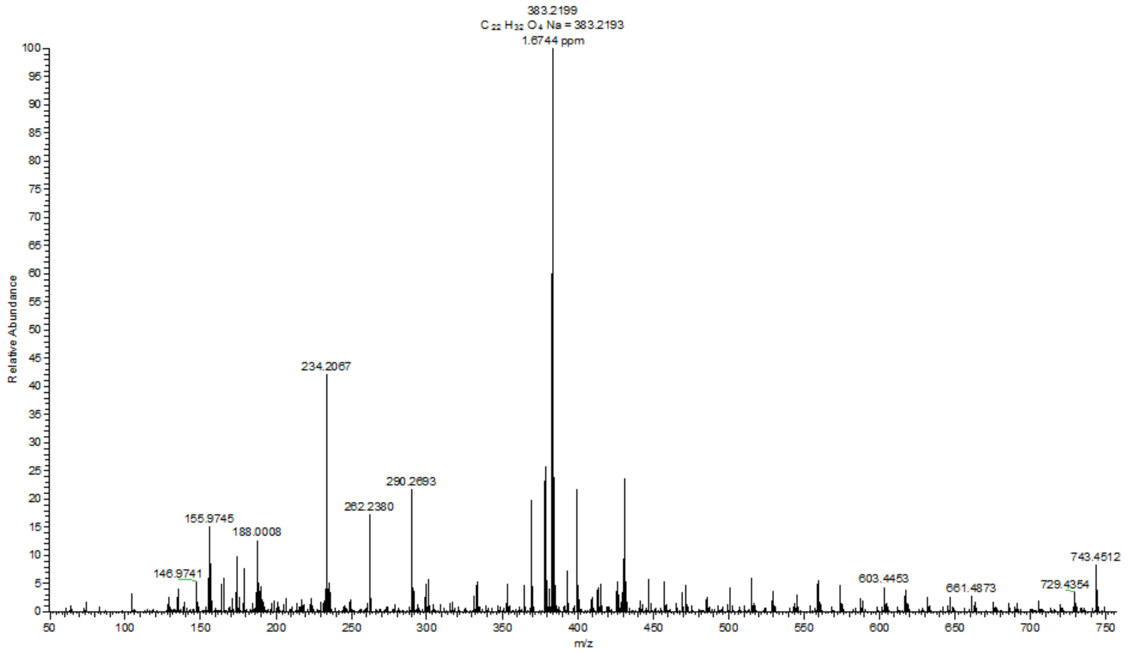

Figure S10. ^{1}H NMR spectrum of dendrillolactone (**1**, $CDCl_3$, 600 MHz).

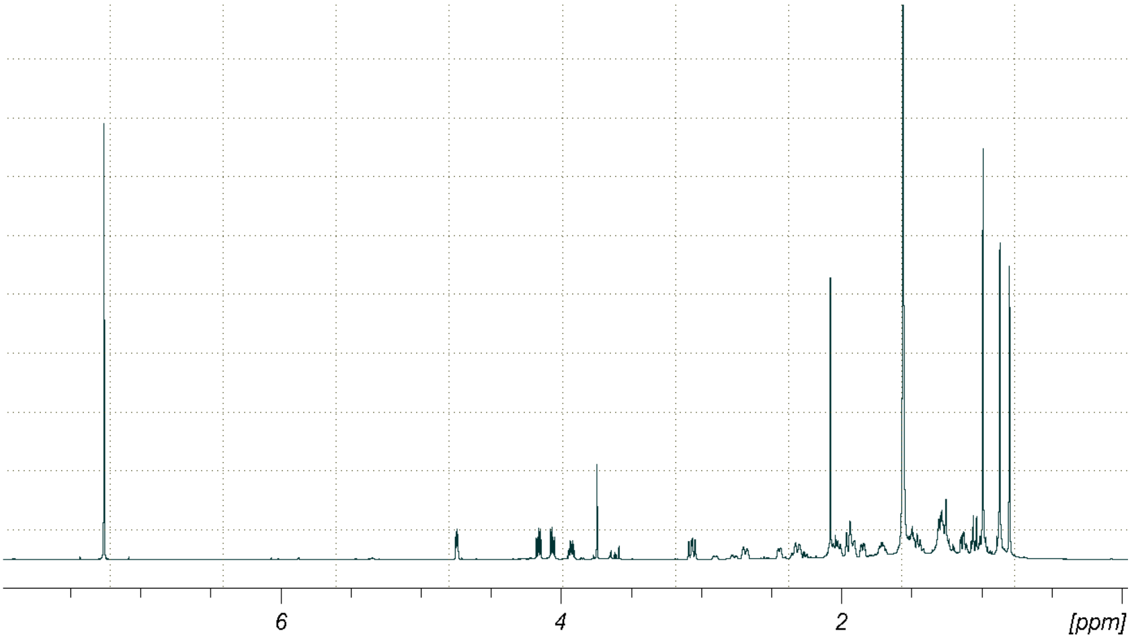

Figure S11. ^{13}C NMR spectrum of dendrillolactone (**1**, $CDCl_3$, 150 MHz).

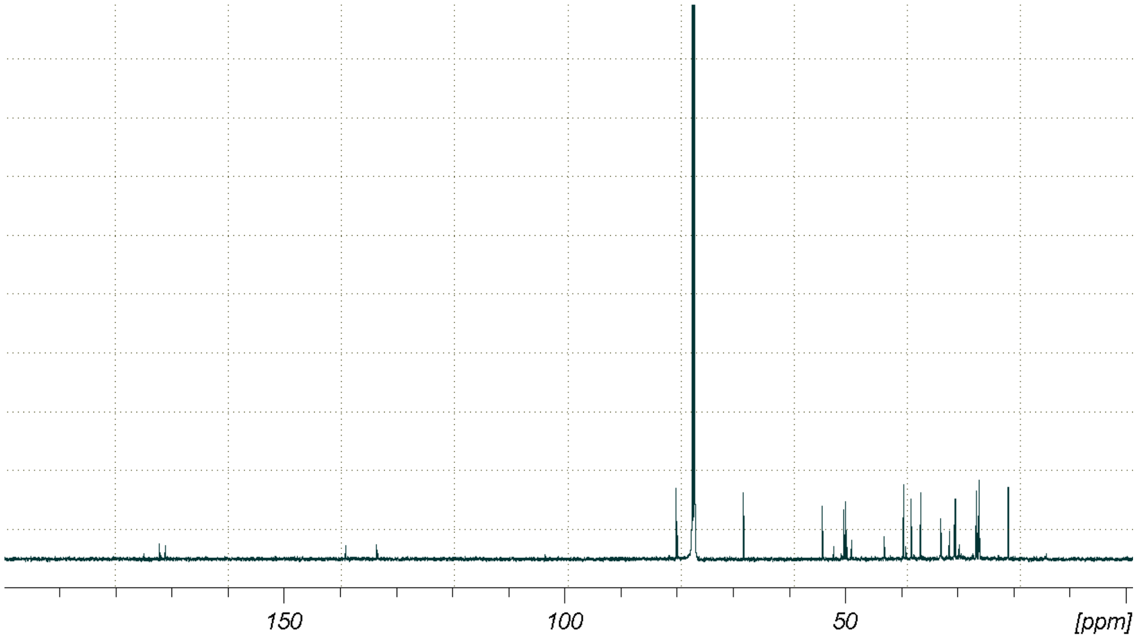

Figure S12. $^{1}H,^{1}H$ COSY NMR spectrum of dendrillolactone (**1**, $CDCl_3$, 600 MHz).

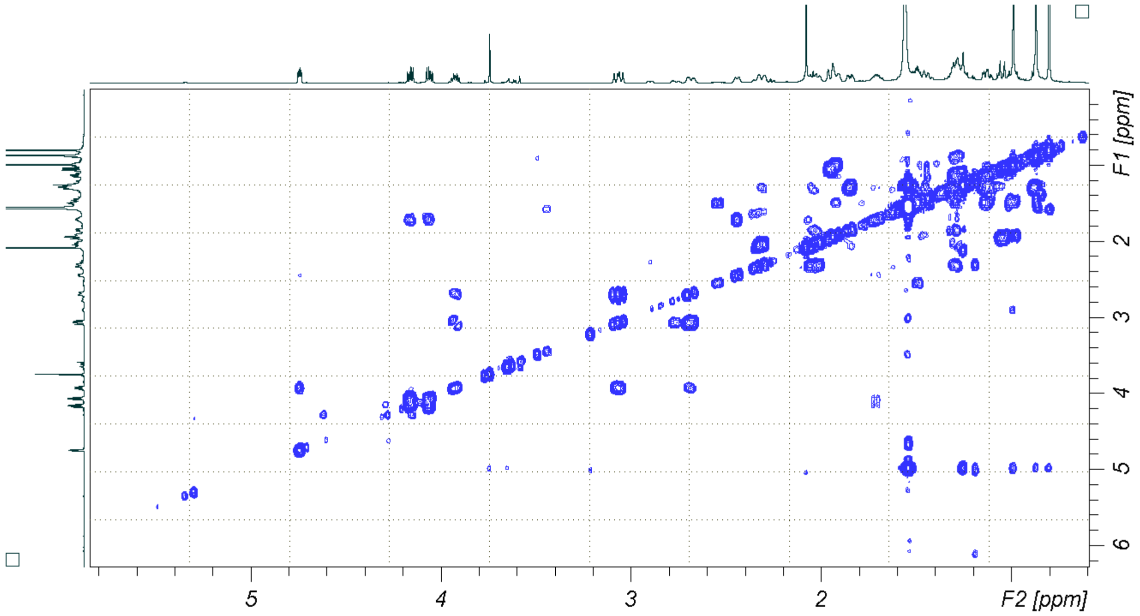

Figure S13. ^{1}H,^{1}H TOCSY NMR spectrum of dendrillolactone (**1**, $CDCl_3$, 600 MHz).

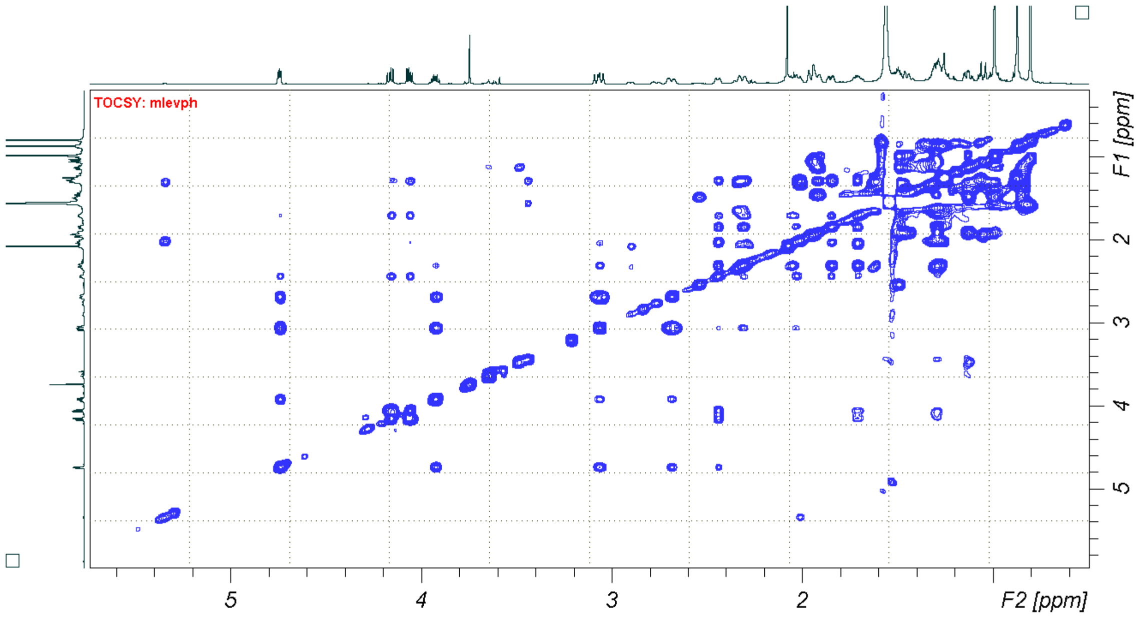

Figure S14. ^{1}H,^{13}C edited-HSQC NMR spectrum of dendrillolactone (**1**, $CDCl_3$, 400 MHz).

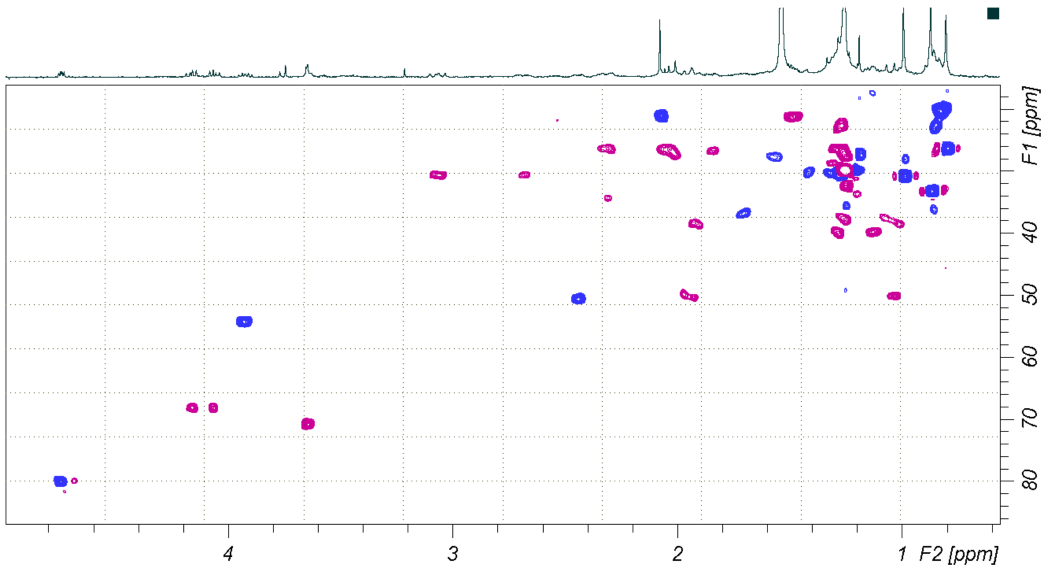

Figure S15. $^{1}H,^{13}C$ HMBC NMR spectrum of dendrillolactone (**1**, $CDCl_3$, 600 MHz).

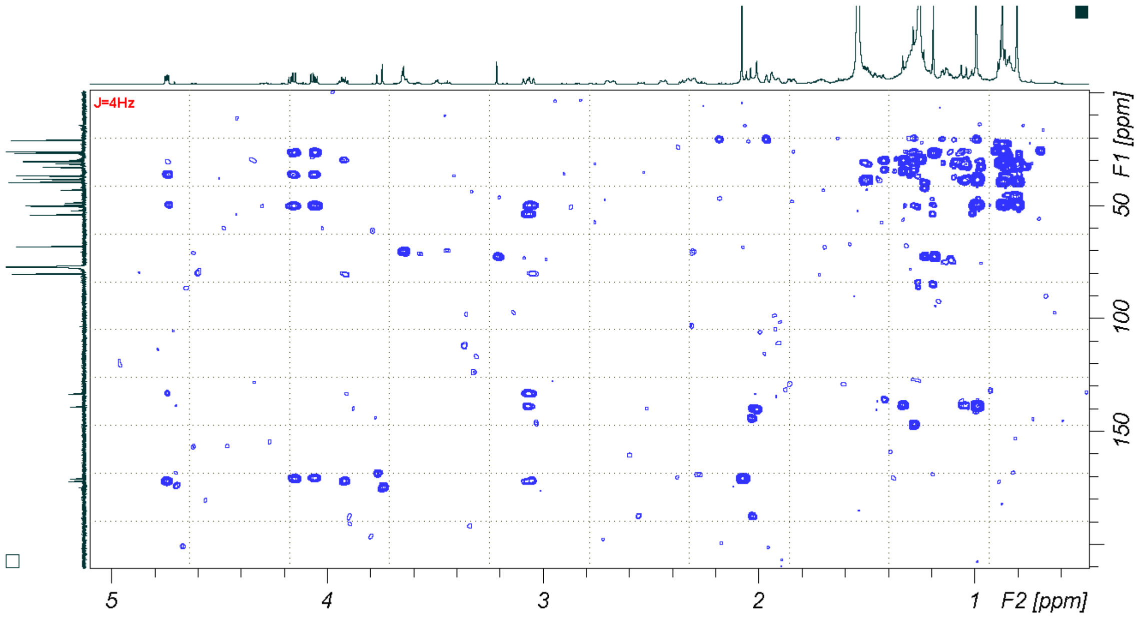

Supplement: Supplementary file 1 [file marinedrugs-23-00010-s001.zip › marinedrugs-3326412-supplementary.pdf]
